# The function of miRNAs in the process of kidney development

**DOI:** 10.1016/j.ncrna.2023.08.009

**Published:** 2023-08-23

**Authors:** Pengfei Sun, Jiaqi Wang, Tatiana Ilyasova, Alina Shumadalova, Murad Agaverdiev, Chunlei Wang

**Affiliations:** a Tianjin Baodi Hospital/Baodi Clinical College of Tianjin Medical University, Tianjin, 301800, China; b Harbin Medical University Cancer Hospital, No. 150 Haping Road, Nangang District, Harbin, 150081, China; c Department of Internal Diseases, Bashkir State Medical University, Ufa, Republic of Bashkortostan 450008, Russia; d Department of General Chemistry, Bashkir State Medical University, 3 Lenin Street, Ufa, Republic of Bashkortostan, 450008, Russia; e Department of Urology, Bashkir State Medical University, 450008, Ufa, Russian Federation; f Department of Neurosurgery, The First Affiliated Hospital of Harbin Medical University, Harbin, 150001, China

**Keywords:** miRNAs, lncRNAs, Kidney development, Kidney dysplasia, Mechanism, Pathology

## Abstract

MicroRNAs (miRNAs) are a class of small non-coding RNAs (ncRNAs) that typically consist of 19–25 nucleotides in length. These molecules function as essential regulators of gene expression by selectively binding to complementary target sequences within messenger RNA (mRNA) molecules, consequently exerting a negative impact on gene expression at the post-transcriptional level. By modulating the stability and translation efficiency of target mRNAs, miRNAs play pivotal roles in diverse biological processes, including the intricate orchestration of organ development. Among these processes, the development of the kidney has emerged as a key area of interest regarding miRNA function. Intriguingly, recent investigations have uncovered a subset of miRNAs that exhibit remarkably high expression levels in the kidney, signifying their close association with kidney development and diseases affecting this vital organ. This growing body of evidence strongly suggests that miRNAs serve as crucial regulators, actively shaping both the physiological processes governing kidney function and the pathological events leading to renal disorders. This comprehensive review aims to provide an up-to-date overview of the latest research progress regarding miRNAs and their involvement in kidney development. By examining the intricate interplay between miRNAs and the molecular pathways driving kidney development, this review seeks to elucidate the underlying mechanisms through which miRNAs exert their regulatory functions. Furthermore, an in-depth exploration of the role played by miRNAs in the occurrence and progression of renal dysplasia will be presented. Renal dysplasia represents a significant developmental anomaly characterized by abnormal kidney tissue formation, and miRNAs have emerged as key players in this pathological process. By shedding light on the intricate network of miRNA-mediated regulatory mechanisms involved in kidney dysplasia, this review aims to provide valuable insights for the diagnosis and research of diseases associated with aberrant kidney development.

## 1 Introduction

Since the discovery of a specific non-coding RNAs (ncRNAs) that can silence the gene function of the nematode *Caenorhabditis elegans*, scientists have made significant progress in studying ncRNAs. Among them, microRNAs (miRNAs) have been the most extensively researched type of ncRNA. To date, nearly 28,000 miRNAs have been reported in almost 200 species [1]. It is estimated that up to half of the transcripts are regulated by miRNAs [2]. The gene expression regulation mediated by miRNAs, which represents a conserved mechanism, has been confirmed to participate in various biological processes such as cell differentiation, apoptosis, tumor initiation, and metastasis [2]. Some highly expressed miRNAs in the kidney are believed to play important roles in renal physiology and pathology, potentially serving as diagnostic markers and therapeutic targets for kidney diseases [3]. Current research focused on kidney development indicates that miRNAs play critical roles in this process. This review summarizes the progress in studying the association between miRNAs and kidney development, exploring their potential roles in kidney development and related disorders.

## 2 MiRNAs biogenesis and functions

MiRNAs are a class of endogenous, non-coding, single-stranded RNA fragments found in eukaryotes. They are approximately 19–25 nucleotides in length. miRNAs exhibit diversity, evolutionary conservation, tissue specificity, and temporal regulation, playing important roles in the developmental processes of various tissues and organs. As regulatory factors, miRNAs are widely present in eukaryotic organisms and function by binding to target mRNAs, thereby participating in gene silencing and translation inhibition [2].

**Fig. 1:**
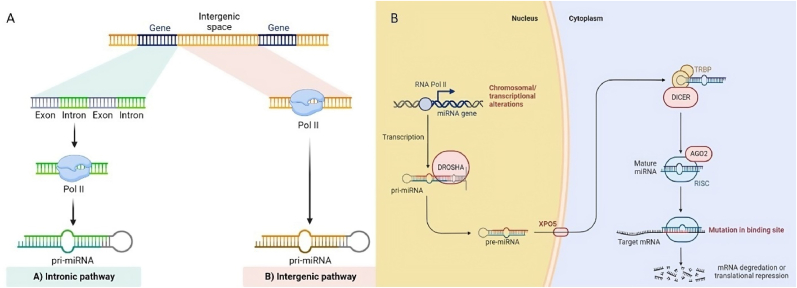
Illustrates the process of microRNA (miRNA) formation and its functional role. (A) MiRNAs are generally classified as intronic or intergenic based upon their genomic location. (B) The miRNA gene is transcribed by RNA polymerase II (RNA Pol II) into a primary miRNA (pri-miRNA) transcript. The Microprocessor complex (DGCR8-Drosha) processes the pri-miRNA into a precursor miRNA (pre-miRNA), which is then exported to the cytoplasm through the transport protein exportin-5 (XPO5). In the cytoplasm, the pre-miRNA is cleaved by Dicer, generating the mature miRNA. The mature miRNA recognizes its target mRNA, recruits the RNA-induced silencing complex (RISC), and mediates post-transcriptional inhibition of the target by translation repression, adenylation, and/or enhanced mRNA degradation.

miRNAs are generally encoded by intergenic DNA sequences. Within the cell nucleus, genomic DNA undergoes transcription by RNA polymerase II (RNA Pol II), resulting in the production of primary miRNA transcripts (pri-miRNAs) that are several thousand base pairs long. The pri-miRNAs are then processed by the Microprocessor complex, composed of RNase III endonuclease Drosha and DGCR8 protein, within the cell nucleus. This processing generates a hairpin structure of approximately 70 nucleotides known as the precursor miRNA (pre-miRNA). Subsequently, the pre-miRNA is transported from the nucleus to the cytoplasm through the action of the RNA-GTP-dependent nuclear-cytoplasmic transport protein exportin-5 (XPO5), forming a complex. After that, the pre-miRNA precursor is transported to the cytoplasm, where it is converted into a mature double-stranded miRNA form by the enzyme ribonuclease III (Dicer) (Fig. 1A–B) [4].

Most miRNAs act as negative regulators of gene expression. miRNAs typically bind to the 3′ untranslated region (3′-UTR) of messenger RNA (mRNA), leading to gene degradation or translation repression. The inhibitory activity of endogenous miRNAs depends on their loading into the RNA-induced silencing complex (RISC). Single-stranded miRNAs are loaded onto the Argonaute (AGO) protein, forming the RISC complex. The complex targets and binds to the complementary 3′-UTR of the mRNA, thereby regulating the expression of the target mRNA [3]. The mode of action of miRNAs depends on their complementarity to the target gene. When miRNAs have perfect complementary pairing with the target mRNA, it can potentially affect the cleavage and degradation of the target mRNA [1]. When there is imperfect pairing between the miRNA and the target mRNA, miRNA can inhibit translation or promote mRNA adenylation and decay, thereby suppressing protein synthesis [5]. In animals, most miRNAs exhibit imperfect pairing with their target mRNAs, which predominantly affects protein expression levels through this mechanism. However, in some cases, certain miRNAs can enhance the translation of specific target mRNAs. For example, miRNAs can form specific complexes by associating with proteins like AGO2, activating the translation of target genes in different cellular states (e.g., G0) [6].

The generation and degradation of miRNAs are tightly regulated to ensure specific miRNAs are expressed at appropriate levels and times in cells. Dysregulation of miRNA expression can lead to uncontrolled downstream target gene expression and contribute to disease development [1]. Current research indicates that miRNA expression is regulated at multiple levels:

(1) Transcriptional regulation: miRNAs located between genes are transcribed from their independent promoters, while miRNAs located within introns can be co-transcribed with their host genes or transcribed independently. The transcription of miRNA is also regulated by transcription factors, enhancers, silencing elements, and chromatin modifications [7]. Approximately 75 transcription factors have been reported to be involved in miRNA transcriptional regulation, with common ones including nuclear factor kappa-light-chain-enhancer of activated B cells (NF-kB), c-Myc, p53, and CCAAT/enhancer binding protein α (C/EBPα) (C/EBPα).
(2) Post-transcriptional regulation: After miRNA genes are transcribed, the entire process from pri-miRNA to mature processing and assembly into the RISC complex is finely regulated. Mechanisms involved in this regulation include RNA editing, regulation of the miRNA microprocessing complex, and RNA-binding proteins specific to certain miRNAs [2]. Key molecules in the miRNA processing pathway, such as Drosha and Dicer, require the formation of complexes with their respective auxiliary molecules to function properly. The expression levels and activities of these molecules are also tightly regulated [2,7].
(3) Degradation regulation: Current studies suggest that miRNA degradation is regulated through modifications such as adenylation or uridylation of miRNA residues, the formation of RNA-protein complexes, and degradation by nucleases [5]. Additionally, a newly discovered pathway for miRNA degradation, called target RNA-directed miRNA degradation (TDMD), has been identified. It involves the specific binding of target RNA to a highly complementary miRNA, leading to the degradation of the bound miRNA [8].
(4) Epigenetic regulation: It is estimated that approximately 50% of miRNA genes are associated with CpG islands, and the expression of many miRNAs is influenced by DNA methylation [9]. Research also indicates that many miRNAs undergo simultaneous methylation and acetylation as part of epigenetic regulation. Recent studies have shown that some miRNAs can also feedback regulate epigenetic mechanisms, highlighting the complexity of miRNA regulatory networks and contributing to the stability of gene regulatory systems [9].

## 3 MiRNAs and kidney development

Mammalian kidneys originate from the intermediate mesoderm as the nephric duct, also known as the pronephros. Kidney development begins in humans at embryonic day 18 (E18) and in mice at embryonic day 8.5 (E8.5). The process of kidney development can be divided into three stages: the pronephros, mesonephros, and metanephros. The pronephros and mesonephros are transient structures that regress during embryonic development, while the metanephros develops into the permanent kidney.

At human E22/mouse E9.5, the anterior part of the nephric duct differentiates into the pronephric tubules. Subsequently, the posterior part of the nephric duct gradually forms the mesonephric tubules. At human E35/mouse E10.5, the caudal end of the mesonephric duct elongates dorsally to form the ureteric bud (UB). As the UB invades the mesenchyme, the nephric duct differentiates into metanephric mesenchyme (MM). The UB and MM interact with each other, promoting the development of the metanephros. The UB undergoes successive branching to form a complete urinary collecting system, while the MM undergoes mesenchymal-epithelial transition. Some MM cells differentiate into non-epithelialized stromal cells, forming smooth muscles, stroma, and the renal microvascular system. Another portion of the MM differentiates to form renal units, including renal corpuscles, proximal and distal convoluted tubules, loops of Henle, and distal tubules.

Overall, this process describes the sequential development of the kidney from the pronephros and mesonephros to the metanephros, involving the differentiation of various cell types and the establishment of the urinary collecting system and renal units [10].

Although there is a growing body of research on miRNAs in the kidney, the available data on the role of miRNAs in kidney development is limited, and their specific functions remain unclear. In recent years, sequencing studies have provided insights into the expression profiles of miRNAs in the embryonic kidneys of mice, which have facilitated research on miRNAs in kidney development [11,12]. Aguilar et al. discovered that miR-199b, miR-25, miR-27b, and miR-200b were highly expressed in the fetal kidneys at embryonic day 12 and 13 (E12 and E13), but their expression was lower in adult kidney tissues [11]. This suggests that miRNA expression in the kidney exhibits temporal differences during kidney development. Moreover, during the transition from E12 to E13 and into adulthood, several miRNAs were downregulated, including miR-17, miR-196a, miR-15b, miR-23b, miR-20a, miR-200c, miR-93, miR-26b, miR-16, miR-218, and 151-5p. On the other hand, miRNAs such as miR-320, miR-351, miR-652, miR-107, miR-103, miR-322, miR-106b, miR-210, miR-125b-5p, miR-199a-5p, and miR-433 were upregulated. Some miRNAs, such as miR-134, miR-152, miR-669b, miR-15a, miR-125-5p, miR-126-5p, miR-99b, and let-7c, showed no significant changes in their expression levels. These miRNAs exhibit temporal differential expression during kidney development and warrant further exploration [11].

A substantial body of research suggests that miRNAs play a regulatory role in coordinating the timing of embryonic development and differentiation [13,14]. A recent study revealed that the Lin28b/let-7 axis, which exhibits temporal differential expression during kidney development, regulates the duration of mouse kidney development by upregulating the insulin-like growth factor-2 (Igf2), a growth-promoting gene involved in kidney morphogenesis [15]. This indicates the potential regulatory role of the time-specific miRNA expression mentioned above in different stages of kidney development.

Furthermore, specific studies have investigated the role of miRNAs in kidney development by selectively knocking out miRNAs or key components of miRNA biogenesis, such as Drosha and Dicer, in kidney tissues/cells [16,17]. These experiments have resulted in a range of kidney defects in developing embryos, including the formation of edema, delayed renal epithelial differentiation, and reduced glomerular number, highlighting the importance of miRNA gene regulation in kidney development directly or indirectly (Table 1).

**Table 1:** MicroRNAs (miRNAs) associated with kidney development in animals.

Species	Tissue/cell specificity	Targets	Mechanism	Reference
African clawed frog (*Xenopus laevis*)	Non-kidney specific	Dicer and Dgcr8	Renal edema, delayed differentiation of renal epithelial cells in the pronephric duct, and abnormal renal morphology	[16]
Mouse	Cells that produce renin	Dicer	Severely reduced number of periglobular cells in adult kidney with renovascular disease and striated fibrosis	[18]
Mouse	Nephron progenitor cells	Dicer	Premature exhaustion of nephron progenitor cells	[19]
Mouse	Collecting duct system of nephron and ureteric buds origin	Dicer	Premature apoptosis of nephron progenitors with defect in UB branching	[20]
Mouse	Renal tubules and ureteric buds	Dicer	Reduced tubular branches, reduced nephrons, bilateral hydronephrosis	[21]
Mouse	Pronephric mesenchyme	Dicer	Ureteric bud branching and failure of nephron progenitor differentiation	[22]
Mouse	Renal progenitor cells and their derivatives	miR-17∼92	The number of nephrons is reduced, and glomerular dysfunction and proteinuric kidney disease develop after birth.	[23]
Mouse	Urogenital and renal tubular system	Dgcr8	Severe hydronephrosis, renal cysts, progressive renal failure within 2 months of birth	[24]
Mouse	Renal stromal cells	Dicer1	Kidney dysplasia with abnormal differentiation of tubules and vasculature	[25]

Studies have shown that miRNAs may participate in early kidney development by influencing key transcription factors. Several transcription factors expressed in renal progenitor cells, including SIX Homeobox 2 (Six2), spalt like transcription factor 1 (Sall1), paired Box gene 2 (Pax2), and Wilms' tumor 1 (WT1), are essential for their proliferation, survival, and subsequent differentiation [[26], [27], [28]]. A study found that eliminating Dicer function in the metanephric mesenchyme resulted in a significant reduction of Six2, Sall1, WT1, Pax2, and Asp/Glu-rich C-terminal domain 1 (CITED1) in renal progenitor cells, accompanied by increased expression of the pro-apoptotic protein Bim in the metanephric mesenchyme, ultimately leading to severe renal developmental defects [22]. Silencing let-7e in embryonic stem cells has been shown to downregulate WT1, Pax2, and Wnt4 [29]. Furthermore, miR-743a has been found to inhibit the proliferation of metanephric mesenchymal stem cells by targeting WT1 in vitro, suggesting its potential role in kidney development and kidney-related diseases [30]. These studies highlight the critical role of miRNAs in regulating the survival of these cell lineages during early kidney organogenesis.

The LIM-class homeobox factor Xlim1/Lhx1 is an important transcription factor required for early renal tubule formation and nephron differentiation. It exhibits a tightly regulated dynamic expression pattern during kidney development [31]. A study on African clawed frog kidney development demonstrated that knockout of miR-30a-5p in the kidney led to delayed differentiation, reduced nephron size, and decreased proliferation [16]. Further investigation revealed that miR-30a-5p targets and inhibits Xlim1/Lhx1. In the absence of miR-30a-5p, Xlim1/Lhx1 remains at high levels, resulting in delayed terminal differentiation of renal epithelial cells [16]. Additionally, Lhx1 interacts cooperatively with the transcriptional coactivator Fryl to regulate early kidney development by modulating the expression of miR-199a/214 and the miR-23b/27b/24a cluster [32]. These studies indicate the indispensable role of miRNAs in regulating early kidney development, particularly in the regulation of early nephrogenesis.

During the process of posterior kidney development, the budding and branching of the ureteric bud are critical steps. The glial-cell-line-derived neurotrophic factor (GDNF)/tyrosine kinase receptor (c-Ret) signaling pathway plays a major role in inducing ureteric bud branching [33]. Studies have found that specific deletion of Dicer in the cells of the nephron lineage and ureteric bud-derived collecting duct system in mice disrupts branch morphogenesis, and the phenotype is associated with downregulation of Wnt11 and c-Ret expression at the tip of the ureteric bud. Therefore, it can be inferred that Dicer regulates the GDNF/c-Ret signaling pathway in mouse kidney development by influencing Dicer-dependent miRNA activity [34]. Previous studies related to neurodevelopment and diseases have shown that miR-9, miR-96, miR-133b, and miR-146a inhibit the expression of GDNF by interacting with its 3′UTR. When these miRNAs replace the less responsive miRNAs and RNA-binding proteins to the 3′UTR sequence of GDNF, it leads to increased endogenous GDNF expression (GDNF hyper) [35]. A recent study found that mice with GDNF hyper/hyper exhibit smaller and malformed kidneys [36], demonstrating that the levels and function of GDNF in kidney development are influenced by its 3′UTR. These studies suggest that miRNAs may participate in kidney development by influencing the GDNF/c-Ret signaling pathway.

Bone morphogenetic proteins (BMPs) are members of the transforming growth factor-β (TGF-β) superfamily of growth factors. They play a crucial role in the normal development of the ureteric bud and nephron formation during kidney development. Mutations in the BMP4 gene can lead to kidney developmental defects. Several recent studies have provided evidence of the interplay between miRNAs and key genes in the TGF-β/BMP signaling pathway.

One mechanism by which the TGF-β/BMP signaling pathway regulates miRNA levels is through the interaction of downstream effector proteins, such as Smad, with Drosha [37]. This interaction facilitates the processing of primary transcripts into mature miR-21 in vascular smooth muscle cells [37]. MiR-21 also plays an important role in the kidney, as it has been reported to promote proliferation and inhibit apoptosis during kidney regeneration in fish [38]. These findings suggest that miR-21 is likely involved in kidney development, possibly through mechanisms beyond the TGF-β/BMP signaling pathway.

Numerous studies have demonstrated that miRNAs participate in the regulation of epithelial-mesenchymal transition (EMT) through the involvement of the TGF-β receptor 2 (TGFβR2). EMT is a critical process in various physiological and pathological events, including kidney fibrosis and embryonic development. It has been confirmed that TGFβR2 is a target of miR-302. Increased expression of miR-302d in mesangial cells leads to reduced expression of TGFβR2 [39]. MiR-590 is another EMT-inhibitory miRNA that targets TGFβR2. Overexpression of miR-590 suppresses EMT by upregulating the epithelial cell marker E-cadherin and downregulating mesenchymal markers such as laminin and α-SMA in human kidney 2 (HK2) cell lines [40]. Additionally, miR-200a directly targets β-catenin in proximal tubular epithelial cells to inhibit TGF-β1-induced EMT [41]. The miR-200 family is highly expressed in early kidneys [16], suggesting that elevated levels of miR-200 may protect renal epithelial cells from spontaneous dedifferentiation during kidney development. Conversely, miR-21 overexpression enhances TGF-β1-induced EMT by inhibiting its target, Smad7 [42]. The let-7b/c has also been shown to suppress TGF-β/Smad signaling activation by downregulating TGFβR1 [43]. These studies collectively indicate the potential involvement of miRNAs in kidney development.

Furthermore, in studies examining the regulation of key molecules involved in kidney fibrosis by miRNAs, it has been found that miR-22 and BMP-7/6 are part of a regulatory feedback loop. MiR-22 not only inhibits the expression of BMP-7/6 but is also induced by BMP-7/6, thereby highlighting the critical role of miR-22 in BMP signaling cascades [44]. Although there is substantial evidence of the interplay between miRNAs and TGF-β/BMP signaling, the specific functions of these miRNAs in developing kidneys remain largely uncertain, providing new directions for future research on the role of miRNAs in kidney development.

The renin-angiotensin system (RAS) is a major regulator of blood pressure and fluid/electrolyte homeostasis, and it plays a central role in controlling normal kidney development [45]. The main components of the RAS system include renin, angiotensinogen, angiotensin-converting enzyme, angiotensin I/II (Ang I/II), and angiotensin II type 1/2 receptor (AT1R and AT2R). All components of the RAS system are highly expressed during kidney development. Sequeira-Lopez et al. [18] generated conditional Dicer knockout mice specifically in renin-producing cells to selectively inhibit miRNA maturation in these cells. Dicer knockout resulted in a severe reduction in juxtaglomerular cell numbers in adult kidneys, accompanied by decreased gene expression of renin 1/2 (Ren1 and Ren2), reduced plasma renin concentration, and the presence of renal functional abnormalities and severe renal vascular defects. This indicates that miRNAs are essential for the specification of renin cells and normal renal vascular development. Furthermore, studies in adult tissues have demonstrated that miRNAs can regulate protein expression at all levels of the RAS cascade [46]. For example, miR-155 in endothelial cells and vascular smooth muscle cells targets and inhibits the expression of AT1R, thereby significantly reducing Ang II-induced signaling [47,48]. This suggests the important role of miRNAs in regulating RAS signaling. However, there are still few reports on specific miRNAs regulating RAS components during kidney development.

Chromatin modification is an epigenetic mechanism that can influence gene transcription activity. Histone deacetylases (HDACs) play important roles in many cellular processes, including cell cycle, proliferation, differentiation, and cell death [49]. Studies in zebrafish and mice have indicated the involvement of HDACs in the development of the pronephros and metanephros. Treatment of zebrafish embryos with HDAC inhibitors resulted in increased numbers of nephron progenitor cells, ultimately leading to impaired kidney function due to excessive nephron progenitor cell proliferation [50]. Culturing E13.5 mouse kidneys with Scriptaid, an inhibitor of class I and class II HDACs, suppressed the expression of transcription factors required for metanephric development, affecting normal cell proliferation and apoptosis and ultimately resulting in impaired kidney development [51]. These studies suggest the critical role of HDACs in regulating kidney development. It has been shown that high glucose can exacerbate the effects of HDAC4 by inhibiting miR-29a signaling, leading to protein deacetylation and degradation in podocytes and ultimately causing renal dysfunction [52]. Another study found that HDAC inhibitor treatment suppressed the expression of calcium transport-related gene Claudin-14 by stimulating the transcription of mouse kidney miR-9 and miR-374 genes, leading to a reduction in urinary calcium excretion in mice [53]. This suggests that the interaction between miRNAs and HDACs and their impact on downstream target genes may play an important role in renal homeostasis. Although there are few specific studies on their mechanisms in kidney development, these pieces of evidence provide a link between miRNAs, HDACs, and kidney development, which warrants further investigation in the future.

## 4 Altered renal MiRNAs expression and abnormal renal development

**Fig. 2:**
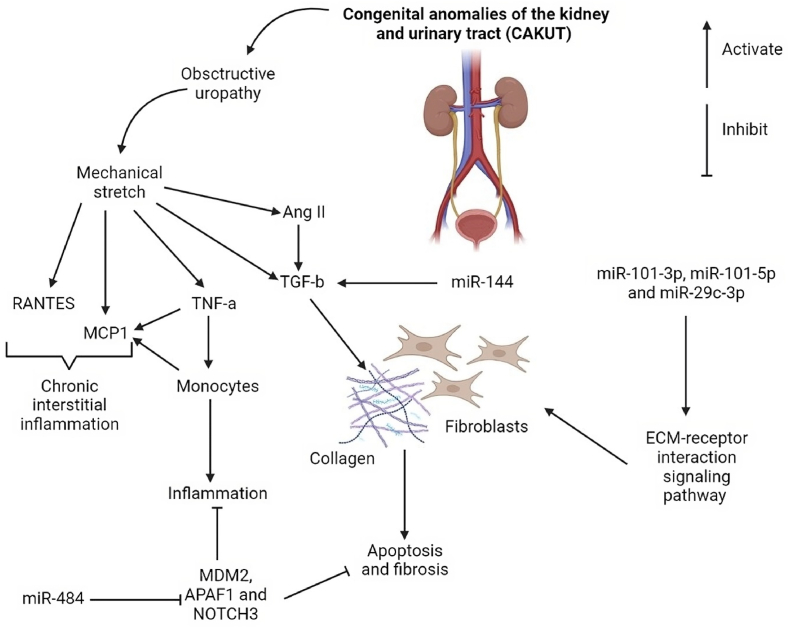
Assigning the congenital anomalies of the kidney and urinary tract (CAKUT)-related biological functions to some microRNAs (miRNAs). Among CAKUT categories, congenital obstructive uropathy represents a common and severe form of malformation. Transforming growth factor beta (TGF-b) and tumor necrosis factor alpha (TNF-α) are well known as central mediators of fibrosis and inflammation and are thought to play an important role in the progression of CAKUT. The increase of monocyte chemoattractant protein-1 (MCP-1) expression levels suggests that the main factor responsible for the above effects is chronic renal inflammation mediated by local monocytes. MiRNAs play an important role in the regulation of these target genes and downstream signaling pathways (RANTES, mouse double minute 2 homolog (MDM2), apoptotic protease activating factor 1 (APAF1), NOTCH3, and extracellular matrix (ECM)-receptor interaction signaling pathways).

As mentioned earlier, several studies have investigated the role of miRNA-mediated gene regulation in kidney development by targeting key enzymes involved in miRNA biogenesis within specific cell lineages of the kidney. The results have revealed that the kidneys of miRNA-deficient animals exhibit various congenital anomalies of the kidney and urinary tract (CAKUT) [21]. Therefore, it raises the question of whether miRNAs play a significant role in the mechanisms underlying fetal kidney developmental abnormalities. This question has garnered increasing attention from researchers in recent years (Fig. 2).

In recent decades, scientific research has provided a deeper understanding of developmental abnormalities in the kidneys. Studies have indicated that genetic variations and changes in the fetal environment are major factors contributing to fetal renal developmental abnormalities [54]. Chromosomal abnormalities, copy number variations, and single-gene genetic abnormalities are the most common factors leading to CAKUT. Currently, population-based and animal studies have identified several genes associated with CAKUT, such as hepatocyte nuclear factor-1 beta (HNF-1β), Pax2, eyes absent homolog 1 (EYA1), SIX5, Ret, Sall1, and WT1 [55]. Among them, autosomal dominant mutations in HNF-1β are the most common monogenic cause of CAKUT and are often associated with renal hypoplasia and non-functioning dysplastic kidneys [56]. Additionally, biallelic gene inactivation mutations in Ret are associated with the most severe manifestation of CAKUT, bilateral renal agenesis [57]. Furthermore, mutations or abnormal expression of Pax2 are frequently observed in renal developmental defects or malformations, and mutations in EYA1/Six1 are associated with branchio-oto-renal syndrome [58].

Another important factor contributing to CAKUT, and delayed kidney development is changes in the fetal environment [59]. Numerous studies have shown that exposure to adverse conditions during pregnancy can affect kidney development, leading to a decrease in the number of nephrons, impaired kidney function, and long-term programming for hypertension and chronic kidney disease in adulthood [60]. These factors include maternal malnutrition, inadequate placental blood supply, gestational diabetes, glucocorticoids, nicotine, alcohol, vitamin A deficiency, and maternal medication exposure (such as angiotensin-converting enzyme inhibitors, antibiotics, phenytoin, antiepileptic drugs, and cyclophosphamide), and their underlying mechanisms have been extensively studied [[61], [62], [63], [64], [65], [66], [67], [68], [69]].

Studies have shown that maternal mice subjected to a low protein diet (LP) during pregnancy can result in intrauterine growth retardation (IUGR) in offspring and exhibit impaired kidney development, possibly associated with RAS inhibition and increased Na+-ATPase activity [61]. A series of animal studies in this experiment have also confirmed that maternal exposure to caffeine, ethanol, nicotine, and dexamethasone during pregnancy can affect the expression of RAS-related genes in the fetal kidneys, leading to impaired kidney development in offspring [64,[70], [71], [72]]. Additionally, we found that maternal caffeine exposure during pregnancy can induce programming of nephrotoxicity in offspring through decreased expression of Kruppel-like factor 4 (KLF4), resulting in increased susceptibility to adult kidney disease [73]. In the IUGR animal model induced by maternal ethanol exposure, alterations in the “Glucocorticoid-insulin-like growth factor 1 (GC-IGF1) axis” programming were found to play a crucial role in impaired kidney development and susceptibility to glomerulosclerosis in adulthood [70]. Moreover, studies have suggested that maternal smoking causes oxidative stress and mitochondrial changes in the kidneys, affecting adult kidney structure, blood pressure, and urinary sodium excretion in offspring [61]. Furthermore, prenatal exposure to dexamethasone can lead to a decrease in the number of nephrons by affecting Wnt4 expression, subsequently influencing TGF-β expression, increasing apoptosis, upregulating pro-apoptotic gene Bax, and downregulating anti-apoptotic gene Bcl-2 [74].

In recent years, numerous studies have shown that miRNA dysregulation is associated with developmental defects in various organisms and organ systems, including the kidney. Some studies have provided evidence of the involvement of miRNAs in the pathogenesis of renal developmental abnormalities.

Genomic sequencing techniques of miRNA genes, facilitated by genetic variations, have contributed to the research on miRNAs in disease. Currently, only a few studies have established a clear link between miRNAs and specific genetic variations in renal diseases. Jovanovic et al. [75] analyzed whole-genome expression data from 19 CAKUT patients and 9 control samples of ureter tissue, identifying 7 miRNAs that potentially play a role in CAKUT: hsa-miR-144, hsa-miR-101, hsa-miR-375, hsa-miR-200a, hsa-miR-183, hsa-miR-495, and hsa-miR-222. Among them, hsa-miR-144 was found to be significantly upregulated in CAKUT patient tissues and may be involved in critical biological processes related to normal kidney and urinary tract development. However, further functional analysis is needed to reveal the role of these specific miRNAs in renal developmental abnormalities. Studies have also shown that the miR-1792 cluster appears to be essential for normal embryonic development and its loss can lead to human developmental disorders such as Feingold syndrome, which includes renal developmental defects [76]. Additionally, several studies have indicated that the miR-1792 cluster is upregulated in various mouse models of polycystic kidney disease (PKD), and its inactivation slows cyst proliferation [77]. This is mainly because the miR-17∼92 cluster targets and inhibits genes associated with cystic kidney diseases, including polycystin 1/2 (Pkd1/2) and HNF-1β. Another miRNA implicated in autosomal dominant polycystic kidney disease is miR-21, which is upregulated in cysts of affected individuals and mice. The potential mechanism by which miR-21 exacerbates cyst growth may involve direct inhibition of the pro-apoptotic tumor suppressor gene programmed cell death 4 (PDCD4) [78]. These studies suggest that miRNAs are key regulators in the pathogenic mechanisms of kidney developmental disorders.

In the context of environmentally induced renal developmental abnormalities, miRNA regulation may also play a crucial role. A recent study found that administration of miRNA inhibitors to pregnant mice resulted in sustained significant reduction in miRNA levels in offspring's kidneys and other organs. This suggests that certain drugs taken during pregnancy that induce miRNA expression, such as tetracycline-based tetracycline-controlled transactivators and tamoxifen-based estrogen receptor systems, can affect miRNA expression in offspring's kidneys through maternal-placental-fetal transmission [79]. Furthermore, an animal study on maternal protein restriction revealed significant downregulation of certain miRNAs in the renal glomeruli of offspring rats (*Rattus norvegicus*), including miR-141 (71%), miR-200a (50%), miR-200b (60%), and miR-429 (59%) [80]. Although these studies did not directly explore the relationship between miRNA dysregulation and developmental abnormalities in offspring's kidneys, they suggest an association between miRNAs and environmentally induced renal developmental disorders. Further research is needed to elucidate the specific roles of more miRNAs in these conditions.

## 5 LNCRNAs/MIRNAs interaction

Long non-coding RNAs (lncRNAs) are more than 200 bases long, transcribed by RNA Pol II, capped and polyadenylated at the 5′ and 3′ ends, respectively [81]. Sequences encoding lncRNAs can be located in intergenic regions, in introns, or partially overlap exons, localizing both on the forward and reverse strands. As a result, they can be divided into five subclasses: sense, antisense, bidirectional, intergenic and intron. LncRNA molecules are involved in various processes: from histone modification and influence on chromatin remodeling to the regulation of transcriptional and post-transcriptional processes. They can be enhancers, scaffolds, “sponges” that compete for binding sites with other RNAs, as well as precursors of some miRNAs [82]. Loss or impairment of kidney function is a common result of several metabolic disorders, including arterial hypertension (AH) and diabetes mellitus (DM). Recent evidence suggests that regulation mechanisms, including lncRNAs-miRNAs-mRNAs interaction, are critical to kidney function as well as disease progression. Basic research has shown that including lncRNAs-miRNAs-mRNAs interaction are involved in kidney development, and their dysregulation can lead to various pathogenic processes, including acute kidney failure (AKI), chronic kidney disease (CKD), and tumor development. Table 2 presents the results of studies that studied the lncRNAs-miRNAs-mRNAs interaction in some kidney diseases [83-.

**Table 2:** Some last studies about long non-coding RNAs (lncRNAs)-microRNAs (miRNAs)-messenger RNAs (mRNAs) interactions in kidney diseases.

Disease	LncRNAs	Expression of lncRNAs	MiRNAs	mRNA targets	Function	References
AKI to CKD	Neat1	Up	miR-129-5p	FADD, CASP-8, and CASP-3	Associated with kidney injury and tubular epithelial cells apoptosis	[83]
Kidney transplant AKI	LncRNA XIST	Up	miR-212-3p and miR-122-5p	ASF1A, BRWD1M, and PFKFB2	Regulate the development of AKI	[84]
RCC	LncRNA-ENTPD3-AS1	Up	miR-155	HIF-1α	Inhibit tumor cell proliferation and tumor development	[85]
ON	MALAT1	Up	miR-145	FAK pathway	Aggravates renal fibrogenesis	[86]
Hyperuricaemia	HOTAIR	Up	miR-22	NLRP3	Promotes endothelial cell pyroptosis	[87]
RCC	LncHILAR	Up	miR-613/206/1-1-3p	Jagged-1/Notch/CXCR4 signaling pathway	Promotes metastasis	[88]
AKI to CKD	LncRNA-H19	Up	miR-196a-5p	Wnt1 and β-catenin	Progression of Fibrosis	[89]
DN	1700020I14Rik	Up	miR-34a-5p	Sirt1 and HIF-1α	Regulates cell proliferation and fibrosis	[90]
ccRCC	COL18A1-AS1	Down	miR-1286	KLF12	Represses tumor progression	[91]
AKI	LncRNA ENSMUST_147219	Up	miR-221-5p	IRF6	Promotes the development of ischemic AKI	[92]

Abbreviations, AKI, Acute kidney injury; CKD, Chronic kidney disease; RCC, Renal cell carcinoma; ON, Obstructive nephropathy; DN, Diabetic nephropathy; ccRCC, Clear cell renal cell carcinoma; Neat1, Nuclear paraspeckle assembly transcript 1; XIST, X-inactive specific transcript; ENTPD3-AS1, ENTPD3 antisense RNA 1; MALAT1, Metastasis-associated lung adenocarcinoma transcript 1; HOTAIR, HOX Transcript Antisense RNA; FADD, Fas-associated protein with death domain; CASP-8, Caspase 8; CASP-3, Caspase 3; ASF1A, Anti-silencing function 1a; BRWD1M, Bromodomain and WD repeat-containing protein 1; PFKFB2, 6-Phosphofructo-2-Kinase/Fructose-2,6-Biphosphatase 2; HIF-1α, Hypoxia-inducible-factor 1A; FAK, Focal adhesion kinase; NLRP3, NLR family pyrin domain containing 3; CXCR4, C-X-C chemokine receptor type 4; Sirt1, Sirtuin 1; KLF12, Krüppel-like factor 12; IRF6, Interferon regulatory factor-6.

## 6 Conclusion

In recent years, there has been a growing interest in exploring the role of miRNAs as essential regulatory molecules in kidney development and diseases. This field of study has garnered significant attention, and researchers are making notable progress in unraveling the complex mechanisms involving miRNAs in the kidney. Numerous studies have demonstrated that miRNAs exhibit distinct expression patterns during kidney development, indicating their active participation in this intricate process. By influencing key growth factors and signaling pathways, these miRNAs play a vital role in orchestrating the precise development and maturation of the kidney. Notably, experiments involving the knockout of critical miRNA processing enzymes, such as Drosha or Dicer, have shed light on the indispensable nature of miRNAs in kidney development. These knockout studies have further emphasized the crucial role played by miRNAs in ensuring the normal growth and functionality of the kidney. However, despite the advancements made, there are still many unanswered questions in this field. One significant challenge arises from the fact that knocking out Drosha or Dicer leads to global changes in miRNA expression, making it challenging to pinpoint the specific functions of individual miRNAs or miRNA clusters in kidney development. Moreover, while the involvement of miRNAs in kidney developmental disorders is increasingly recognized, the precise mechanisms underlying their contribution to these disorders remain elusive. Further research is needed to unravel the intricate interplay between miRNAs and the molecular pathways implicated in kidney developmental disorders. To advance our understanding, future research should focus on deciphering the precise roles of individual miRNAs or groups of miRNAs in kidney development. It is essential to uncover the intricate mechanisms through which these miRNAs regulate physiological processes during kidney development and how dysregulation can lead to pathological conditions. To facilitate such investigations, it is crucial to harness the power of advanced sequencing technologies. These technologies can provide comprehensive profiles of miRNA expression and facilitate the identification of key miRNAs that are critically involved in kidney developmental disorders. Constructing miRNA-related gene networks specific to kidney development will be instrumental in unraveling the complex interactions and regulatory networks underlying normal kidney development and related diseases. This comprehensive understanding holds the potential to not only identify early biomarkers for kidney diseases but also provide valuable insights into therapeutic targets that could revolutionize the treatment and management of kidney disorders. In conclusion, miRNAs have emerged as crucial regulators in kidney development and diseases. Despite the existing challenges and unanswered questions, ongoing research efforts are paving the way for a deeper understanding of the roles and mechanisms of miRNAs in kidney development. By leveraging advanced technologies and interdisciplinary approaches, researchers aim to unlock the full potential of miRNAs as diagnostic tools and therapeutic targets in the field of kidney diseases.

## Author contributions

Pengfei Sun and Jiaqi Wang wrote the draft and revised it. Pengfei Sun and Jiaqi Wang designed and supervised the study. Tatiana Ilyasova, Alina Shumadalova, Murad Agaverdiev, Chunlei Wang collected the data and designed the figures and tables. All the authors read the submitted version and approved it.

## Funding

This work was supported by the Bashkir State Medical University Strategic Academic Leadership Program (PRIORITY-2030).

## Declaration of competing interest

The authors declare that they have no conflict of interests.

## References

[1] Saliminejad K., Khorram Khorshid H.R., Soleymani Fard S., Ghaffari S.H. (2019). An overview of microRNAs: biology, functions, therapeutics, and analysis methods. J. Cell. Physiol..

[2] Beylerli O., Gareev I., Sufianov A., Ilyasova T., Guang Y. (2022). Long noncoding RNAs as promising biomarkers in cancer. Noncoding RNA Res..

[3] Sufianov A., Begliarzade S., Beilerli A., Liang Y., Ilyasova T., Beylerli O. (2022). Circular RNAs as biomarkers for lung cancer. Noncoding RNA Res..

[4] Ma S.Y., Bai Y., Han N., Wang J.H., Weng X.Y., Bian H.W., Zhu M.Y. (2012). Recent research progress of biogenesis and functions of miRNA. Hereditas.

[5] Valadkhan S., Gunawardane L.S. (2013). Role of small nuclear RNAs in eukaryotic gene expression. Essays Biochem..

[6] Sufianov A., Kostin A., Begliarzade S., Kudriashov V., Ilyasova T., Liang Y., Mukhamedzyanov A., Beylerli O. (2023). Exosomal non coding RNAs as a novel target for diabetes mellitus and its complications. Noncoding RNA Res..

[7] Kawahara Y., Zinshteyn B., Chendrimada T.P., Shiekhattar R., Nishikura K. (2007). RNA editing of the microRNA-151 precursor blocks cleavage by the Dicer-TRBP complex. EMBO Rep..

[8] Gareev I., Kudriashov V., Sufianov A., Begliarzade S., Ilyasova T., Liang Y., Beylerli O. (2022). The role of long non-coding RNA ANRIL in the development of atherosclerosis. Noncoding RNA Res..

[9] Zhang W.T., Duan N., Zhang Q., Song T., Li Z., Zhang C.G., Chen X., Wang K.Z. (2017). DNA methylation mediated down-regulation of miR-370 regulates cell growth through activation of the wnt/β-catenin signaling pathway in human osteosarcoma cells. Int. J. Biol. Sci..

[10] Schedl A. (2000). Hastie ND. Cross-talk in kidney development. Curr. Opin. Genet. Dev..

[11] Aguilar A.L.G., Piskol R., Beitzinger M., Zhu J.Y., Kruspe D., Aszodi A., Moser M., Englert C., Meister G. (2010). The small RNA expression profile of the developing murine urinary and reproductive systems. FEBS Lett..

[12] Nagalakshmi V.K., Lindner V., Wessels A., u J. (2015). microRNA-dependent temporal gene expression in the ureteric bud epithelium during mammalian kidney development. Dev. Dyn..

[13] Ambros V. (2011). MicroRNAs and developmental timing. Curr. Opin. Genet. Dev..

[14] Schulman B.R.M., Esquela-Kerscher A., Slack F.J. (2005). Reciprocal expression of lin-41 and the microRNAs let-7 and mir-125 during mouse embryogenesis. Dev. Dyn..

[15] Yermalovich A.V., Osborne J.K., Sousa P., Han A., Kinney M.A., Chen M.J., Robinton D.A., Montie H., Pearson D.S., Wilson S.B., Combes A.N., Little M.H., Daley G.Q. (2019). Lin28 and let-7 regulate the timing of cessation of murine nephrogenesis. Nat. Commun..

[16] Agrawal R., Tran U., Wessely O. (2009). The miR-30 miRNA family regulates Xenopus pronephros development and targets the transcription factor Xlim1/Lhx1. Development.

[17] Cerqueira D.M., Bodnar A.J., Phua Y.L., Freer R., Hemker S.L., Walensky L.D., Hukriede N.A., Ho J. (2017). Bim gene dosage is critical in modulating nephron progenitor survival in the absence of microRNAs during kidney development. FASEB J..

[18] Sequeira-Lopez M.L.S., Weatherford E.T., Borges G.R., Monteagudo M.C., Pentz E.S., Harfe B.D., Carretero O., Sigmund C.D., Gomez R.A. (2010). The microRNA-processing enzyme dicer maintains juxtaglomerular cells. J. Am. Soc. Nephrol..

[19] Ho J., Pandey P., Schatton T., Sims-Lucas S., Khalid M., Frank M.H., Hartwig S., Kreidberg J.A. (2011). The pro-apoptotic protein bim is a microRNA target in kidney progenitors. J. Am. Soc. Nephrol..

[20] Nagalakshmi V.K., Ren Q., Pugh M.M., Valerius M.T., McMahon A.P., Yu J. (2011). Dicer regulates the development of nephrogenic and ureteric compartments in the mammalian kidney. Kidney Int..

[21] Bartram M.P., Höhne M., Dafinger C., Völker L.A., Albersmeyer M., Heiss J., Göbel H., Brönneke H., Burst V., Liebau M.C., Benzing T., Schermer B., Müller R.U. (2013). Conditional loss of kidney microRNAs results in congenital anomalies of the kidney and urinary tract (CAKUT). J. Mol. Med..

[22] Chu J.Y.S., Sims-Lucas S., Bushnell D.S., Bodnar A.J., Kreidberg J.A., Ho J. (2014). Dicer function is required in the metanephric mesenchyme for early kidney development. Am. J. Physiol. Ren. Physiol..

[23] Marrone A.K., Stolz D.B., Bastacky S.I., Kostka D., Bodnar A.J., Ho J. (2014). MicroRNA-17∼92 is required for nephrogenesis and renal function. J. Am. Soc. Nephrol..

[24] Bartram M.P., Dafinger C., Habbig S., Benzing T., Schermer B., Müller R.U. (2015). Loss of Dgcr8-mediated microRNA expression in the kidney results in hydronephrosis and renal malformation. BMC Nephrol..

[25] Nakagawa N., Xin C.Y., Roach A.M., Naiman N., Shankland S.J., Ligresti G., Ren S.Y., Szak S., Gomez I.G., Duffield J.S. (2015). Dicer1 activity in the stromal compartment regulates nephron differentiation and vascular patterning during mammalian kidney organogenesis. Kidney Int..

[26] Kreidberg J.A., Sariola H., Loring J.M., Maeda M., Pelletier J., Housman D., Jaenisch R. (1993). WT-1 is required for early kidney development. Cell.

[27] Rothenpieler U.W., Dressler G.R. (1993). Pax-2 is required for mesenchyme-to-epithelium conversion during kidney development. Development.

[28] Dressler G.R., Patel S.R. (2015). Epigenetics in kidney development and renal disease. Transl. Res..

[29] Viñas J.L., Ventayol M., Brüne B., Jung M., Sola A., Pi F., Mastora C., Hotter G. (2013). miRNA let-7e modulates the Wnt pathway and early nephrogenic markers in mouse embryonic stem cell differentiation. PLoS One.

[30] Xue M.M., Zhou Y.R., Liu X.Y., Ni D.S., Hu Y.X., Long Y.S., Ju P., Zhou Q. (2016). Proliferation of metanephric mesenchymal cells is inhibited by miR-743a-mediated WT1 suppression in vitro. Mol. Med. Rep..

[31] Dressler G.R. (2006). The cellular basis of kidney development. Annu. Rev. Cell Dev. Biol..

[32] Espiritu E.B., Crunk A.E., Bais A., Hochbaum D., Cervino A.S., Phua Y.L., Butterworth M.B., Goto T., Ho J., Hukriede N.A., Cirio M.C. (2018). The Lhx1-Ldb1 complex interacts with Furry to regulate microRNA expression during pronephric kidney development. Sci. Rep..

[33] Majumdar A., Vainio S., Kispert A., McMahon J., McMahon A.P. (2003). Wnt11 and Ret/Gdnf pathways cooperate in regulating ureteric branching during metanephric kidney development. Development.

[34] Maheu M., Lopez J.P., Crapper L., Davoli M.A., Turecki G., Mechawar N. (2015). MicroRNA regulation of central glial cell line-derived neurotrophic factor (GDNF) signalling in depression. Transl. Psychiatry.

[35] Kumar A., Kopra J., Varendi K., Porokuokka L.L., Panhelainen A., Kuure S., Marshall P., Karalija N., Härma M.A., Vilenius C., Lilleväli K., Tekko T., Mijatovic J., Pulkkinen N., Jakobson M., Jakobson M., Ola R., Palm E., Lindahl M., Strömberg I., Võikar V., Piepponen T.P., Saarma M., Andressoo J.O. (2015). GDNF overexpression from the native locus reveals its role in the nigrostriatal dopaminergic system function. PLoS Genet..

[36] Li H., Jakobson M., Ola R., Gui Y.J., Kumar A., Sipilä P., Sariola H., Kuure S., Andressoo J.O. (2019). Development of the urogenital system is regulated via the 3'UTR of GDNF. Sci. Rep..

[37] Davis B.N., Hilyard A.C., Lagna G., Hata A. (2008). SMAD proteins control DROSHA-mediated microRNA maturation. Nature.

[38] Hoppe B., Pietsch S., Franke M., Engel S., Groth M., Platzer M., Englert C. (2015). MiR-21 is required for efficient kidney regeneration in fish. BMC Dev. Biol..

[39] Faherty N., Curran S.P., O'Donovan H., Martin F., Godson C., Brazil D.P., Crean J.K. (2012). CCN2/CTGF increases expression of miR-302 microRNAs, which target the TGFβ type II receptor with implications for nephropathic cell phenotypes. J. Cell Sci..

[40] Liu T.M., Nie F., Yang X.G., Wang X.Y., Yuan Y., Lv Z.S., Zhou L., Peng R., Ni D.S., Gu Y.P., Zhou Q., Weng Y.G. (2015). MicroRNA-590 is an EMT-suppressive microRNA involved in the TGFβ signaling pathway. Mol. Med. Rep..

[41] Gong Y., Qin Z.X., Zhou B.S., Chen H., Shi Z.M., Zhang J. (2017). MicroRNA-200a inhibits transforming growth factor β1-induced proximal tubular epithelial-mesenchymal transition by targeting β-catenin. Nephron.

[42] Wang J.Y., Gao Y.B., Zhang N., Zou D.W., Wang P., Zhu Z.Y., Li J.Y., Zhou S.N., Wang S.C., Wang Y.Y., Yang J.K. (2014). miR-21 overexpression enhances TGF-β1-induced epithelial-tomesenchymal transition by target smad7 and aggravates renal damage in diabetic nephropathy. Mol. Cell. Endocrinol..

[43] Choi H.I., Park J.S., Kim D.H., Kim C.S., Bae E.H., Ma S.K., Kim S.W. (2019). PGC-1α suppresses the activation of TGF-β/Smad signaling via targeting TGFβRI downregulation by let-7b/c upregulation. Int. J. Mol. Sci..

[44] Long J.Y., Badal S.S., Wang Y., Chang B.H.J., Rodriguez A., Danesh F.R. (2013). MicroRNA-22 is a master regulator of bone morphogenetic protein-7/6 homeostasis in the kidney. J. Biol. Chem..

[45] Yosypiv I.V. (2020).

[46] Butterworth M.B. (2018). Role of microRNAs in aldosterone signaling. Curr. Opin. Nephrol. Hypertens..

[47] Stankovic A., Kolaković A., Živković M., Djurić T., Bundalo M., Končar I., Davidović L., Alavantić D. (2016). Angiotensin receptor type 1 polymorphism A1166C is associated with altered AT1R and miR-155 expression in carotid plaque tissue and development of hypoechoic carotid plaques. Atherosclerosis.

[48] Zheng L., Xu C.C., Chen W.D., Shen W.L., Ruan C.C., Zhu L.M., Zhu D.L., Gao P.J. (2010). MicroRNA-155 regulates angiotensin II type 1 receptor expression and phenotypic differentiation in vascular adventitial fibroblasts. Biochem. Biophys. Res. Commun..

[49] Seto E., Yoshida M. (2014). Erasers of histone acetylation: the histone deacetylase enzymes. Cold Spring Harbor Perspect. Biol..

[50] de Groh E.D., Swanhart L.M., Cosentino C.C., Jackson R.L., Dai W.X., Kitchens C.A., Day B.W., Smithgall T.E., Hukriede N.A. (2010). Inhibition of histone deacetylase expands the renal progenitor cell population. J. Am. Soc. Nephrol..

[51] Chen S.W., Bellew C., Yao X., Stefkova J., Dipp S., Saifudeen Z., Bachvarov D., El-Dahr S.S. (2011). Histone deacetylase (HDAC) activity is critical for embryonic kidney gene expression, growth, and differentiation. J. Biol. Chem..

[52] Lin C.L., Lee P.H., Hsu Y.C., Lei C.C., Ko J.Y., Chuang P.C., Huang Y.T., Wang S.Y., Wu S.L., Chen Y.S., Chiang W.C., Reiser J., Wang F.S. (2014). MicroRNA-29a promotion of nephrin acetylation ameliorates hyperglycemia-induced podocyte dysfunction. J. Am. Soc. Nephrol..

[53] Gong Y.F., Himmerkus N., Plain A., Bleich M., Hou J.H. (2015). Epigenetic regulation of microRNAs controlling CLDN14 expression as a mechanism for renal calcium handling. J. Am. Soc. Nephrol..

[54] Nicolaou N., Renkema K.Y., Bongers E.M.H.F., Giles R.H., Knoers N.V.A.M. (2015). Genetic, environmental, and epigenetic factors involved in CAKUT. Nat. Rev. Nephrol..

[55] Bertram J.F., Goldstein S.L., Pape L., Schaefer F., Shroff R.C., Warady B.A. (2016). Kidney disease in children: latest advances and remaining challenges. Nat. Rev. Nephrol..

[56] Avni F.E., Lahoche A., Langlois C., Garel C., Hall M., Vivier P.H. (2015). Renal involvement in children with HNF1β mutation: early sonographic appearances and long-term follow-up. Eur. Radiol..

[57] Skinner M.A., Safford S.D., Reeves J.G., Jackson M.E., Freemerman A.J. (2008). Renal aplasia in humans is associated with RET mutations. Am. J. Hum. Genet..

[58] Weber S., Moriniere V., Knüppel T., Charbit M., Dusek J., Ghiggeri G.M., Jankauskiené A., Mir S., Montini G., Peco-Antic A., Wühl E., Zurowska A.M., Mehls O., Antignac C., Schaefer F., Salomon R. (2006). Prevalence of mutations in renal developmental genes in children with renal hypodysplasia: results of the ESCAPE study. J. Am. Soc. Nephrol..

[59] Juvet C., Simeoni U., Yzydorczyk C., Siddeek B., Armengaud J.B., Nardou K., Juvet P., Benahmed M., Cachat F., Chehade H. (2018). Effect of early postnatal nutrition on chronic kidney disease and arterial hypertension in adulthood: a narrative review. J. Dev. Origins Health Dis..

[60] Brophy P. (2017). Maternal determinants of renal mass and function in the fetus and neonate. Semin. Fetal Neonatal Med..

[61] Dötsch J., Alejandre-Alcazar M., Janoschek R., Nüsken E., Weber L.T., Nüsken K.D. (2016). Perinatal programming of renal function. Curr. Opin. Pediatr..

[62] Ergaz Z., Avgil M., Ornoy A. (2005). Intrauterine growth restriction-etiology and consequences: what do we know about the human situation and experimental animal models?. Reprod. Toxicol..

[63] Corrêa R.R.M., Pucci K.R.M., Rocha L.P., Júnior C.D.P., Helmo F.R., Machado J.R., Rocha L.B., Rodrigues A.R.A., Glória M.A., Guimarães C.S.O., Câmara N.O.S., Reis M.A. (2014). Acute kidney injury and progression of renal failure after fetal programming in the offspring of diabetic rats. Pediatr. Res..

[64] Li B., Zhu Y.N., Chen H.Y., Gao H., He H.Y., Zuo N., Pei L.G., Xie W., Chen L.B., Ao Y., Wang H. (2019). Decreased H3K9ac level of AT2R mediates the developmental origin of glomerulosclerosis induced by prenatal dexamethasone exposure in male offspring rats. Toxicology.

[65] Stangenberg S., Nguyen L.T., Chen H., Al-Odat I., Killingsworth M.C., Gosnell M.E., Anwer A.G., Goldys E.M., Pollock C.A., Saad S. (2015). Oxidative stress, mitochondrial perturbations and fetal programming of renal disease induced by maternal smoking. Int. J. Biochem. Cell Biol..

[66] Gray S.P., Denton K.M., Cullen-McEwen L., Bertram J.F., Moritz K.M. (2010). Prenatal exposure to alcohol reduces nephron number and raises blood pressure in progeny. J. Am. Soc. Nephrol..

[67] Goodyer P., Kurpad A., Rekha S., Muthayya S., Dwarkanath P., Iyengar A., Philip B., Mhaskar A., Benjamin A., Maharaj S., Laforte D., Raju C., Phadke K. (2007). Effects of maternal vitamin A status on kidney development: a pilot study. Pediatr. Nephrol..

[68] Rosenblum S., Pal A., Reidy K. (2017). Renal development in the fetus and premature infant. Semin. Fetal Neonatal Med..

[69] Luyckx V.A., Brenner B.M. (2015). Birth weight, malnutrition and kidney-associated outcomes--a global concern. Nat. Rev. Nephrol..

[70] Chen H.Y., Zhu Y.N., Zhao X.Q., He H.Y., Luo J.S., Ao Y., Wang H. (2020). Prenatal ethanol exposure increased the susceptibility of adult offspring rats to glomerulosclerosis. Toxicol. Lett..

[71] Ao Y., Sun Z.X., Hu S.S., Zuo N., Li B., Yang S.L., Xia L.P., Wu Y., Wang L.L., He Z., Wang H. (2015). Low functional programming of renal AT2R mediates the developmental origin of glomerulosclerosis in adult offspring induced by prenatal caffeine exposure. Toxicol. Appl. Pharmacol..

[72] Sun Z.X., Hu S.S., Zuo N., Yang S.L., He Z., Ao Y., Wang H. (2015). Prenatal nicotine exposure induced GDNF/c-Ret pathway repression-related fetal renal dysplasia and adult glomerulosclerosis in male offspring. Toxicol. Res..

[73] Zhu Y.N., Chen H.Y., Zhao X.Q., Li B., He H.Y., Cheng H., Wang H., Ao Y. (2019). Decreased H3K9ac level of KLF4 mediates podocyte developmental toxicity induced by prenatal caffeine exposure in male offspring rats. Toxicol. Lett..

[74] Sheen J.M., Yu H.R., Tiao M.M., Chen C.C., Huang L.T., Chang H.Y., Tain Y.L. (2015). Prenatal dexamethasone-induced programmed hypertension and renal programming. Life Sci..

[75] Jovanovic I., Zivkovic M., Kostic M., Krstic Z., Djuric T., Kolic I., Alavantic D., Stankovic A. (2016). Transcriptome-wide based identification of miRs in congenital anomalies of the kidney and urinary tract (CAKUT) in children: the significant upregulation of tissue miR-144 expression. J. Transl. Med..

[76] de Pontual L., Yao E., Callier P., Faivre L., Drouin V., Cariou S., Van Haeringen A., Geneviève D., Goldenberg A., Oufadem M., Manouvrier S., Munnich A., Vidigal J.A., Vekemans M., Lyonnet S., Henrion-Caude A., Ventura A., Amiel J. (2011). Germline deletion of the miR-17∼92 cluster causes skeletal and growth defects in humans. Nat. Genet..

[77] Patel V., Williams D., Hajarnis S., Hunter R., Pontoglio M., Somlo S., Igarashi P. (2013). miR-17∼92 miRNA cluster promotes kidney cyst growth in polycystic kidney disease. Proc. Natl. Acad. Sci. U.S.A..

[78] Lakhia R., Hajarnis S., Williams D., Aboudehen K., Yheskel M., Xing C., Hatley M.E., Torres V.E., Wallace D.P., Patel V. (2016). MicroRNA-21 aggravates cyst growth in a model of polycystic kidney disease. J. Am. Soc. Nephrol..

[79] Hönig J., Mižíková I., Nardiello C., Solaligue D.E.S., Daume M.J., Vadász I., Mayer K., Herold S., Günther S., Seeger W., Morty R.E. (2020). Transmission of microRNA antimiRs to mouse offspring via the maternal-placental-fetal unit. RNA.

[80] de Barros Sene L., Mesquita F.F., de Moraes L.N., Santos D.C., Carvalho R., Gontijo J.A.R., Boer P.A. (2013). Involvement of renal corpuscle microRNA expression on epithelial-to-mesenchymal transition in maternal low protein diet in adult programmed rats. PLoS One.

[81] Gareev I., Gileva Y., Dzidzaria A., Beylerli O., Pavlov V., Agaverdiev M., Mazorov B., Biganyakov I., Vardikyan A., Jin M., Ahmad A. (2021). Long non-coding RNAs in oncourology. Noncoding RNA Res..

[82] Beylerli O., Gareev I., Pavlov V., Chen X., Zhao S. (2020). The role of long noncoding RNAs in the biology of pituitary adenomas. World Neurosurg..

[83] Ma T., Li H., Liu H., Peng Y., Lin T., Deng Z., Jia N., Chen Z., Wang P. (2022). Neat1 promotes acute kidney injury to chronic kidney disease by facilitating tubular epithelial cells apoptosis via sequestering miR-129-5p. Mol. Ther..

[84] Cheng Q., Wang L. (2020). LncRNA XIST serves as a ceRNA to regulate the expression of ASF1A, BRWD1M, and PFKFB2 in kidney transplant acute kidney injury via sponging hsa-miR-212-3p and hsa-miR-122-5p. Cell Cycle.

[85] Wang J., Zou Y., Du B., Li W., Yu G., Li L., Zhou L., Gu X., Song S., Liu Y., Zhou W., Xu B., Wang Z. (2021). SNP-mediated lncRNA-ENTPD3-AS1 upregulation suppresses renal cell carcinoma via miR-155/HIF-1α signaling. Cell Death Dis..

[86] Liu P., Zhang B., Chen Z., He Y., Du Y., Liu Y., Chen X. (2020). m6A-induced lncRNA MALAT1 aggravates renal fibrogenesis in obstructive nephropathy through the miR-145/FAK pathway. Aging.

[87] Chi K., Geng X., Liu C., Zhang Y., Cui J., Cai G., Chen X., Wang F., Hong Q. (2021). LncRNA-HOTAIR promotes endothelial cell pyroptosis by regulating the miR-22/NLRP3 axis in hyperuricaemia. J. Cell Mol. Med..

[88] Hu G., Ma J., Zhang J., Chen Y., Liu H., Huang Y., Zheng J., Xu Y., Xue W., Zhai W. (2021). Hypoxia-induced lncHILAR promotes renal cancer metastasis via ceRNA for the miR-613/206/1-1-3p/Jagged-1/Notch/CXCR4 signaling pathway. Mol. Ther..

[89] Dong X., Cao R., Li Q., Yin L. (2022). The long noncoding RNA-H19 mediates the progression of fibrosis from acute kidney injury to chronic kidney disease by regulating the miR-196a/Wnt/β-Catenin signaling. Nephron.

[90] Li A., Peng R., Sun Y., Liu H., Peng H., Zhang Z. (2018). LincRNA 1700020I14Rik alleviates cell proliferation and fibrosis in diabetic nephropathy via miR-34a-5p/Sirt1/HIF-1α signaling. Cell Death Dis..

[91] Liu Y., Wang J., Shou Y., Xu W., Huang Z., Xu J., Chen K., Liu J., Liu D., Liang H., Yang H., Zhang X. (2022). Restoring the epigenetically silenced lncRNA COL18A1-AS1 represses ccRCC progression by lipid browning via miR-1286/KLF12 axis. Cell Death Dis..

[92] Liu J., Li X., Yang J., Zhang D. (2022). LncRNA ENSMUST_147219 mediates the progression of ischemic acute kidney injury by targeting the miR-221-5p/IRF6 axis. Apoptosis.

